# Longitudinal changes in the bioactive proteins in human milk of the Chinese population: A systematic review

**DOI:** 10.1002/fsn3.2061

**Published:** 2020-12-19

**Authors:** Qiqi Ren, Yalin Zhou, Wei Zhang, Yueyue Tian, Han Sun, Xuejun Zhao, Yajun Xu, Shilong Jiang

**Affiliations:** ^1^ PKUHSC‐China Feihe Joint Research Institute of Nutrition and Healthy Lifespan Development Beijing China; ^2^ Nutrition and Metabolism Research Division, Innovation Center Heilongjiang Feihe Dairy Co., Ltd. Beijing China; ^3^ Department of Nutrition and Food Hygiene, School of Public Health Peking University Beijing China; ^4^ Beijing Key Laboratory of Toxicological Research and Risk Assessment for Food Safety Peking University Beijing China; ^5^ Present address: Shanghai Institute for Pediatric Research, Xinhua Hospital Shanghai Jiao Tong University School of Medicine Shanghai China

**Keywords:** breast milk, composition, dynamic, profile

## Abstract

This systematic review aimed at investigating longitudinal changes in human milk bioactive protein concentrations in Chinese population. Both English and Chinese databases were searched. The data were pooled into six defined lactation stages. Weighted means of protein concentrations in each stage and the statistical significance of means of different lactation stages were calculated. The data of 11 bioactive proteins were retrieved. Concentrations of sIgA, IgM, and IgG decreased sharply during the first 14 days of lactation. The levels of α‐lactalbumin, lactoferrin, and β‐casein also decreased throughout lactation. Conversely, lysozyme levels increased over lactation. The changing patterns of the serum albumin, osteopontin, and bile salt‐stimulated lipase (BSSL) were not conclusive. This study represents the most comprehensive summary of bioactive proteins in Chinese human milk. In the future, mass spectrometry‐based analysis of human milk proteomics may be used to investigate the longitudinal changes of many more bioactive proteins.

## Graphical abstract

This systematic review aims at investigating the longitudinal changes in the bioactive proteins in the human milk of the Chinese population. The concentrations of α‐lactalbumin, lactoferrin, β‐casein, and three immunoglobulins (sIgA, IgM, and IgG) decrease during lactation. Lysozyme concentrations increase during lactation.

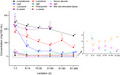

## 1 INTRODUCTION

Human milk provides infants with advantages in cognitive development, defense against pathogens, digestion and absorption of nutrients, lower risk of chronic diseases in later life, etc. (Lönnerdal, 2016b). Accordingly, the World Health Organization recommends that infants should be exclusively breastfed for the first 6 months of their lives. Breastfeeding beyond 6 months—with the addition of appropriate complementary foods—is also recommended (WHO, 2003). The components in human milk contain a complex matrix of nutrients, including proteins and amino acids, lipids, lactose and oligosaccharides, vitamins, minerals, and other substances.

The proteins in human milk are beneficial to infants in two distinct ways. First, amino acids derived from proteins can be assimilated by infants as their tissue proteins, catabolized as metabolic fuels, or converted to intermediate metabolites such as ornithine and citrulline (Kalhan & Bier, 2008). Second, some proteins exhibit beneficial effects on infants as intact proteins or partially digested products (peptides). These proteins are commonly defined as bioactive proteins (Lönnerdal, 2013). Bioactive proteins offer a wide variety of functions such as facilitating nutrient digestion and absorption, modulating immune functions, and defense against pathogens. Table 1 lists bioactive proteins with known or proposed functions (Artym & Zimecki, 2013; Haschke et al., 2016; Lönnerdal, 2016a). Furthermore, it should be noted that the advancement of analytical tools has allowed an increasing number of bioactive proteins to be identified and quantified in human milk.

**Table 1:** List of bioactive proteins investigated in this study

Item	Molecular weight (kDa)	Compartment in human milk	Digestibility by infant's gut	Functions
α‐Lactalbumin	14	Whey	Partial digestion	Zn & Fe absorption; immunomodulation; prebiotics
Lactoferrin	80	Whey	No or limited digestion; intact proteins found in stool	Fe absorption; Immunomodulation; antimicrobial activity; intestinal development; prebiotics; cognitive development
Serum albumin	67	Whey	Easily digested	Unclear
Secretory IgA (sIgA)	60	Whey	No or limited digestion; intact proteins found in stool	Immunomodulation; antimicrobial activity
IgM	74	Whey	Easily digested	Immunomodulation
IgG	50	Whey	Easily digested	Immunomodulation
Lysozyme	14	Whey	No or limited digestion; intact proteins found in stool	Antimicrobial activity
Osteopontin	44–75	Whey	Partial digestion	Immunomodulation
Bile salt‐stimulated lipase (BSSL)	90	Whey	No or limited digestion; intact proteins found in stool	Lipid digestion and absorption; antimicrobial activity
Haptocorrin	60	Whey	No or limited digestion; intact proteins found in stool	Vitamin B12 absorption; antimicrobial activity
Milk fat globule membrane protein (MFGMP)	N/A	Mucin	N/A	Antimicrobial activity, prebiotics
β‐Casein	24	Casein	Partial digestion	Ca, Zn, and P absorption
κ‐Casein	19	Casein	Partial digestion	Antimicrobial activity

Abbreviation: N/A, data not available.

Different bioactive proteins in human milk follow different changing patterns throughout lactation (Lönnerdal et al., 2017). It is possible that the changing patterns of bioactive proteins meet specific needs of the infants during different stages of growth and development. For example, lactoferrin is a bioactive protein that can inhibit bacterial growth and has immunomodulatory properties (Legrand, 2016; Yin et al., 2014). Its concentration is the highest in colostrum, which is consistent with the fact that infants are more vulnerable to foreign pathogens during the first week of life when compared with the rest of infancy (Levy, 2007). Therefore, understanding the changing patterns throughout lactation can shed light on the physiological functions of bioactive proteins.

Genetic and dietary factors may lead to differences in the composition of human milk among different countries (Stam et al., 2013). China has a population of more than 1.4 billion. The bioactive proteins in human milk in Chinese population have been investigated and published in original research articles in both English and Chinese. However, to our best knowledge, there is currently no systematic review that compiles the data of bioactive proteins in Chinese human milk. Compared with original research articles, systematic reviews can provide a more comprehensive summary.

The aim of this study is to investigate the longitudinal changes of human milk bioactive proteins in Chinese population. To achieve this goal, a systematic review was conducted and the statistical significance of bioactive protein levels between different lactation stages was analyzed. Our study represents the first systematic review to summarize the bioactive proteins in human milk in Chinese population.

## 2 METHODS

### 2.1 Literature screening

The PRISMA guidelines were followed (Shamseer et al., 2015). For articles published in English, the databases Pubmed, Web of Science, Taylor & Francis Online, and Springer were searched. The searching strategy of "(human OR breast) AND (milk) AND (bioactive protein OR α‐lactalbumin OR lactoferrin OR secretory IgA OR IgG OR IgM OR lysozyme OR bile salt‐stimulated lipase OR haptocorrin OR osteopontin OR β‐casein OR κ‐casein OR MFGM) AND (composition OR concentration OR content) AND (China OR Chinese)" was used. For articles published in Chinese, the databases China National Knowledge Infrastructure (CNKI; http://cnki.net/), Wanfang Data (http://www.wanfangdata.com.cn), and Chongqing VIP Information (http://qikan.cqvip.com/) were searched. Per the style of the Chinese language, the searching strategy was optimized to "(human OR breast) AND (milk) AND (effective components OR bioactive protein OR α‐lactalbumin OR lactoferrin OR secretory IgA OR IgG OR IgM OR lysozyme OR bile salt‐stimulated lipase OR haptocorrin OR osteopontin OR β‐casein OR κ‐casein OR MFGM) AND (composition OR concentration OR content)." The literature searching was completed in April 2020.

Duplicates and obviously irrelevant articles were removed after reading the titles and abstracts. The full texts of the remaining articles were screened using the inclusion and exclusion criteria listed in PICOS (Table 2). Furthermore, a quality assessment was performed for all the included articles (Table S1). The literature screening process was performed independently by two investigators (Q. R. and Y. Z.). Discrepancies were discussed in the presence of Y. X. and S. J. until consensuses were reached.

**Table 2:** Inclusion and exclusion criteria for selecting articles (PICOS)

Parameter	Inclusion criteria	Exclusion criteria
Population	Chinese population; healthy mothers	Non‐Chinese populations; non‐human; mothers or infants with defined diseases or disorders (premature delivery was not regarded as diseases or disorders)
Intervention	N/A	N/A
Comparator	N/A	N/A
Outcomes	Human milk samples; data were expressed as means or medians; lactation stages could fit into the categories of 1–7, 8–14, 15–30, 31–60, 61–90, 91–365 postnatal days	Lactation stage not specified or simply described as colostrum, transition milk, or mature milk
Study design	Original articles from peer‐reviewed journals; master theses or doctoral dissertations that reported original research data	Review articles; abstracts; articles without access to full‐text; milk samples were pooled together before assessment

### 2.2 Data extraction and analysis

The means and medians were extracted, and the medians were converted to means as previously described (Hozo et al., 2005; Luo et al., 2015). All units were converted to mg/100 ml. The density of the human milk was assumed to be 103.2 g/100 ml for unit conversion (Neville et al., 1988). Lactation was divided into six stages—1–7, 8–14, 15–30, 31–60, 61–90, and beyond 91 postnatal days—and data within each lactation stage were pooled. Outliers were identified using Q3 + 1.5 * (Q3 − Q1) and Q1 − 1.5 * (Q3 − Q1) as upper and lower fences, respectively, and were removed from further calculation. After data cleaning, the weighted means and standard deviations (SDs) were calculated. One‐way ANOVA analyses followed by Student–Newman–Keuls tests were used to determine the statistical significance of the means of each bioactive protein in different lactation stages. Excel and SPSS (19.0) were used for data extraction and analysis. Data extraction and analysis were performed independently by two investigators (Q. R. and Y. Z.). Discrepancies were discussed in the presence of Y. X. and S. J. until consensuses were researched.

## 3 RESULTS

**FIGURE 1:**
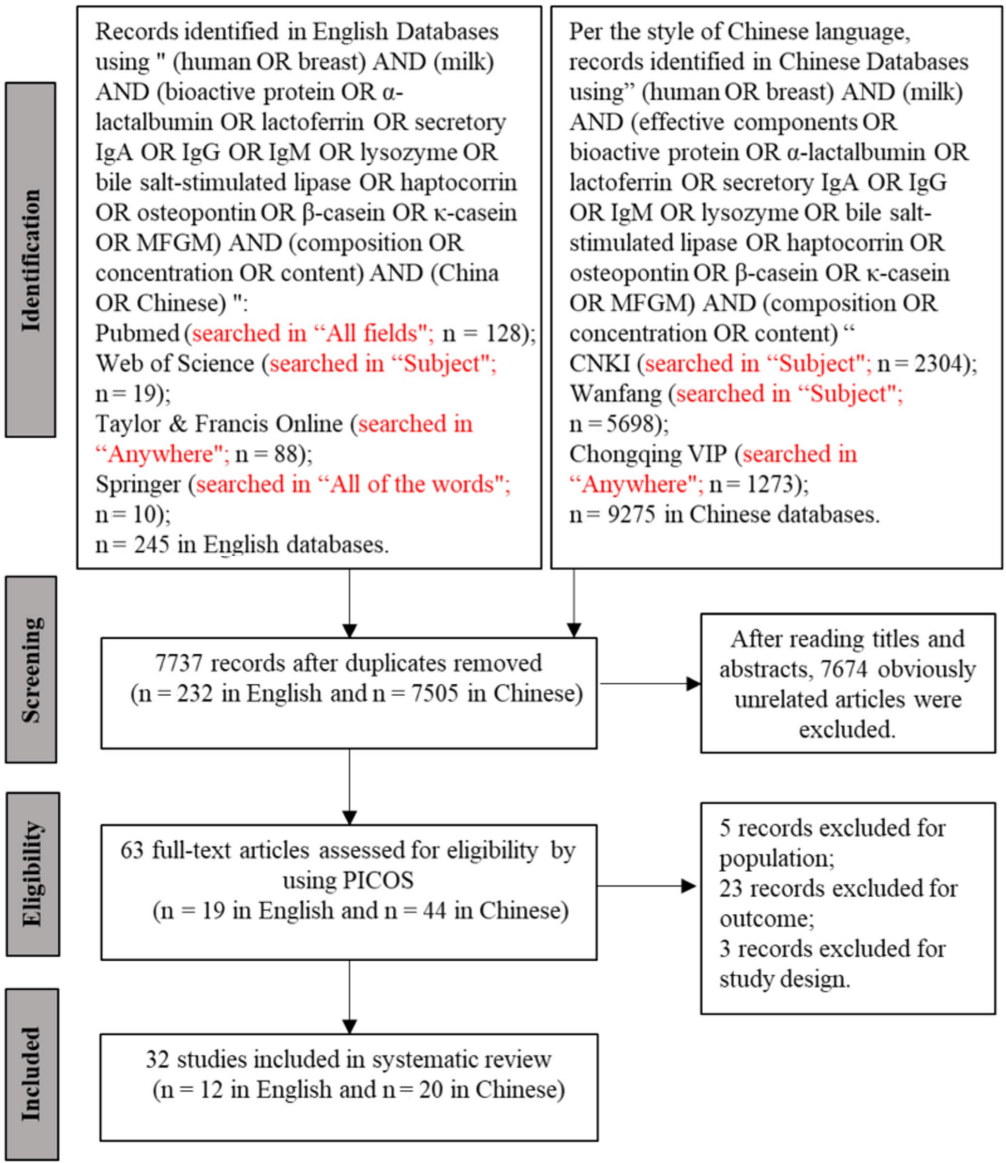
Flow diagram of the literature search process

The literature screening process is shown in Figure 1. The included studies are listed in Table 3 (Affolter et al., 2016; Bruun et al., 2018; Cai et al., 2018; Chen, 2007; Chen et al., 1986; Dai & Guan, 1985; Dou et al., 1986; Elwakiel et al., 2019; Han et al., 2010; Hsu et al., 2014; Jackson et al., 2004; Jiang, 2017; Jiang et al., 1999; Li, et al., 1995; Li, et al., 1995; Liu et al., 1994, 2018, 2019; Liu & Zhang, 2005; Min, 1989; Sha et al., 2019; Shan et al., 2011; Shi et al., 2011; Urwin et al., 2013; Wang et al., 2012; Wang, 1987, 2012; Wang & Lin, 1997; Wei & Pan, 1991; Wu et al., 1995; Yang et al., 2018; Yuen et al., 2012). The included studies covered 23 out of 34 provinces in China. Furthermore, 20 of the included studies specified that milk from mothers who delivered full‐term infants was investigated, whereas the remaining 12 did not indicate the gestation age at delivery. Three of the included studies specified that milk was collected from vaginal delivery mothers, whereas the rest of the studies did not mention the types of delivery. Two studies collected foremilk, four studies collected full expression from one side, and the rest did not specify the means of milk collection (Table 3).

**Table 3:** List of all included studies

Reference	Bioactive proteins reported	Quantification methods	Human milk collection location	Term/preterm	Mode of delivery	Foremilk/hindmilk/full expression
Dai and Guan (1985)	sIgA, IgG, IgM	Single radil immunodiffusion	Hubei	Term	N.S.	N.S.
Wang et al. (2012)	sIgA	ELISA	Inner Mongolia, Shanghai	Term	N.S.	N.S.
Wang (2012)	sIgA	Turbidimetric inhibition immuno assay	Hainan	Term	N.S.	N.S.
Dou et al. (1986)	Lactoferrin, sIgA, IgG, IgM, lysozyme	Single radil immunodiffusion (lactoferrin, sIgA, IgG, IgM); agar plate method (lysozyme)	Shanxi	Term	N.S.	N.S.
Min (1989)	sIgA, IgG, IgM	Single radil immunodiffusion	Hubei	Term	N.S.	N.S.
Li, Guo, et al. (1995)	sIgA, IgG, IgM	Single radil immunodiffusion	Gansu	Term	Vaginal delivery	N.S.
Chen (2007)	sIgA, IgG, IgM	Turbidimetric inhibition immuno assay	Chongqing	Term	N.S.	N.S.
Liu et al. (1994)	sIgA, IgG, IgM	Single radil immunodiffusion	Guangdong	Term	Vaginal delivery	N.S.
Li, Mei, et al. (1995)	sIgA, IgG, IgM	Single radil immunodiffusion	Hebei	Term	Vaginal delivery	N.S.
Wang and Lin (1997)	Lysozyme	Agar plate method	Hubei	N.S.	N.S.	N.S.
Wang (1987)	sIgA	Single radil immunodiffusion	N.S.	N.S.	N.S.	N.S.
Liu and Zhang (2005)	sIgA, IgG, IgM	Single radil immunodiffusion	Liaoning	Term	N.S.	N.S.
Wu et al. (1995)	sIgA, IgG, IgM	Single radil immunodiffusion	Hebei	Term	N.S.	N.S.
Wei and Pan (1991)	Lysozyme	Agar plate method	Jiangsu	N.S.	N.S.	N.S.
Liu et al. (2018)	Lactoferrin	ELISA	Beijing	N.S.	N.S.	Full expression
Jiang (2017)	β‐Casein, α‐lactalbumin	HPLC‐MS	Zhejiang, Gansu, Beijing	N.S.	N.S.	Full expression
Shan et al. (2011)	Lactoferrin	ELISA	Shanghai	Term	N.S.	Foremilk
Jiang et al. (1999)	sIgA, IgG, IgM	Turbidimetric inhibition immuno assay	Guangdong	Term	N.S.	N.S.
Chen et al. (1986)	sIgA	Turbidimetric inhibition immuno assay	Sichuan	Term	N.S.	N.S.
Han et al. (2010)	sIgA, IgG	Radioimmunoassay	Henan	N.S.	N.S.	N.S.
Jackson et al. (2004)	α‐Lactalbumin	HPLC‐MS	Sichuan	Term	N.S.	N.S.
Hsu et al. (2014)	Lactoferrin, sIgA, lysozyme	ELISA	Taiwan	Term	N.S.	N.S.
Liu et al. (2019)	β‐Casein, κ‐casein, α‐lactalbumin, lactoferrin, serum albumin	HPLC‐MS	Shandong, Hubei, Inner Mongolia	N.S.	N.S.	N.S.
Yang et al. (2018)	Lactoferrin	HPLC‐MS	Beijing, Gansu, Guangdong, Guangxi, Heilongjiang, Inner Mongolia, Shandong, Shanghai, Xinjiang, Yunnan, Zhejiang	Term	N.S.	Full expression
Urwin et al. (2013)	sIgA	ELISA	Jiangsu, Shandong, Hebei	N.S.	N.S.	Foremilk
Cai et al. (2018)	Lactoferrin	HPLC‐MS	Beijing, Shanghai, Guangdong, Heilongjiang, Zhejiang	N.S.	N.S.	N.S.
Yuen et al. (2012)	Lactoferrin, sIgA, lysozyme	ELISA	Hongkong	Term	N.S.	N.S.
Bruun et al. (2018)	Osteopontin	ELISA	Hunan	N.S.	N.S.	N.S.
Affolter et al. (2016)	α‐Lactalbumin, Lactoferrin, serum albumin, sIgA, IgG, IgM	LabChip GX‐II (lactoferrin, serum albumin); ELISA (sIgA, IgG, IgM)	Beijing, Jiangsu, Guangdong	Term	N.S.	Full expression
Shi et al. (2011)	α‐Lactalbumin, lactoferrin, serum albumin, sIgA, IgG, IgM	The MDQ capillary electrophoresis system	Inner Mongolia	N.S.	N.S.	N.S.
Sha et al. (2019)	BSSL	ELISA	Jiangsu	N.S.	N.S.	N.S.
Elwakiel et al. (2019)	α‐Lactalbumin, lactoferrin, serum albumin, osteopontin, BSSL, β‐casein, κ‐casein	LC‐MS	Inner Mongolia	Term	N.S.	N.S.

Abbreviation: N.S., not specified.

We retrieved concentrations of 11 bioactive proteins: α‐lactalbumin (6 studies; 5 stages), lactoferrin (11 studies; 6 stages), serum albumin (4 studies; 5 stages), sIgA (20 studies; 6 stages), IgM (12 studies; 6 stages), IgG (13 studies; 6 stages), lysozyme (5 studies; 6 stages), osteopontin (2 studies; 2 stages), BSSL (2 studies; 2 stages), β‐casein (3 studies; 3 stages), and κ‐casein (2 studies; 2 stages). Four proteins accounted for more than 10% of total human milk proteins at any of the lactation stages: α‐lactalbumin, β‐casein, lactoferrin, and sIgA. α‐lactalbumin and β‐casein were the most abundant bioactive proteins, whereas sIgA represented the dominant immune globulin (Table S2).

**FIGURE 2:**
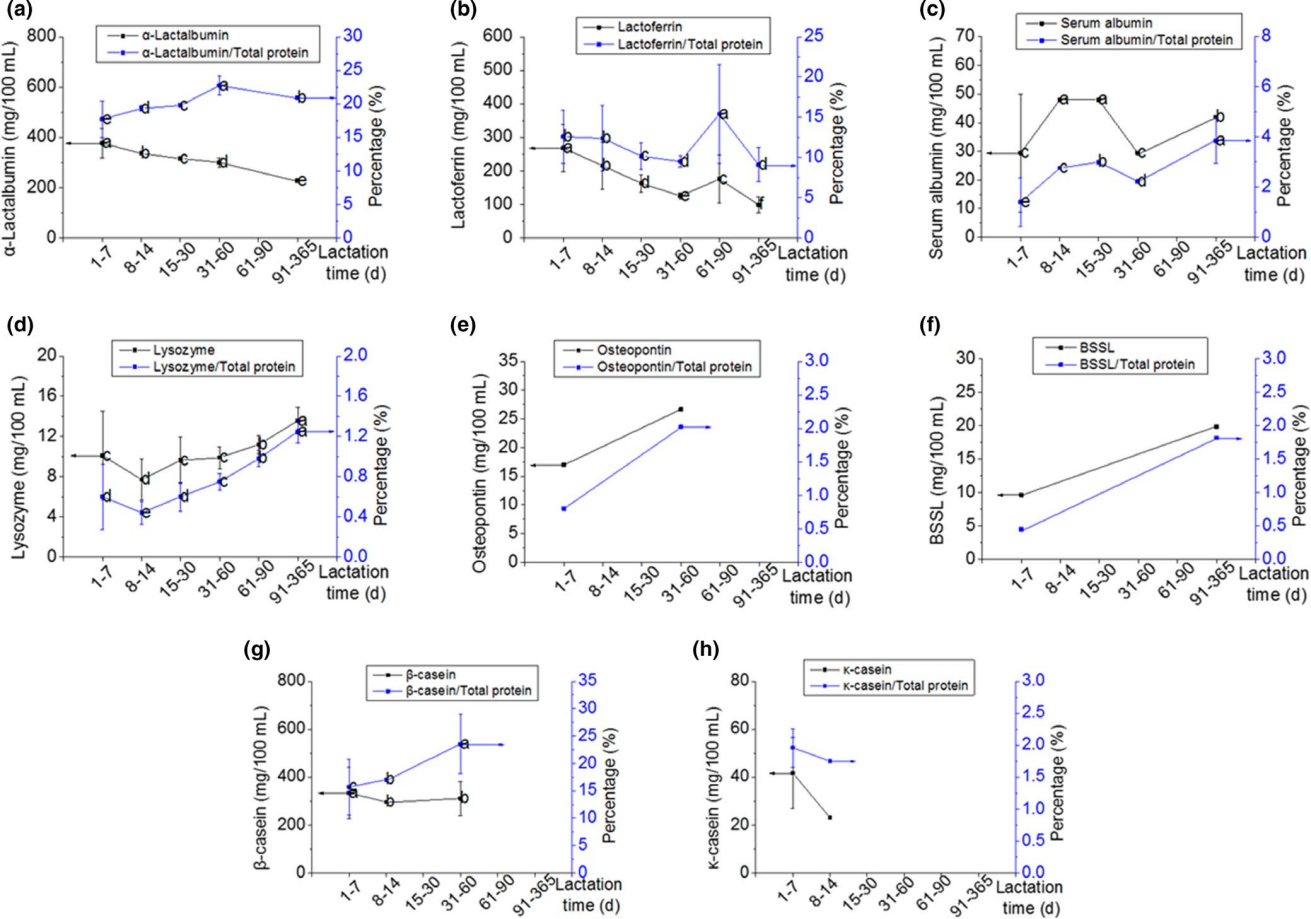
The longitudinal changes in the protein concentrations and the percentages of the total proteins of the bioactive proteins in the human milk of the Chinese population

Concentrations of 5 bioactive proteins decreased significantly during lactation (Figure 2). The levels of α‐lactalbumin and lactoferrin decreased gradually without reaching plateaus throughout lactation, except for the level of lactoferrin during the 61–90 postnatal period, which was significantly higher than either the preceding or subsequent stages. The levels of immunoglobulins were the highest during the first 7 postnatal days, decreased thereafter, and reached plateaus. For sIgA and IgM, the plateaus were reached at the 15–30 postnatal day stage. For IgG, the plateau was reached at the 8–14 postnatal day stage. The concentrations of sIgA, IgM, and IgG during the 1–7 postnatal days stage were 3, 3.5, and 16 times higher than the levels during the 8–14 postnatal day stage (Table S2).

The lysozyme levels were similar during the first 60 postnatal days—except for the 8–14 postnatal day stage—and increased gradually during the remainder of lactation (Figure 2). No clear pattern could be observed in the levels of serum albumin, whose concentrations were higher in the 8–14 and 15–30 postnatal day periods than during the preceding or subsequent stages. For bioactive proteins with data of less than four stages available, the levels of β‐casein and κ‐casein were higher during the first 7 postnatal days than in later stages. Conversely, the levels of osteopontin and BSSL in the first 7 postnatal days were lower than those in later lactation stages.

The percentages of each bioactive protein in total protein generally followed the trends in protein concentration in milk, except for α‐lactalbumin and β‐casein (Figure 2). The percentage of α‐lactalbumin in total protein increased during lactation, which was contradictory to the change in its concentration in human milk. The percentage of β‐casein in total protein during the first 7 postnatal days was lower than those in later stages, whereas its concentration in human milk was higher in the first 7 postnatal days when compared with later stages.

## 4 DISCUSSION

This study represents the most comprehensive overview of bioactive protein concentrations in the human milk of the Chinese population to date. We found that α‐lactalbumin, lactoferrin, sIgA, IgM, and IgG decreased whereas lysozyme increased throughout lactation. The trends of the rest of the included proteins are less conclusive due to a lack of data in the literature. Furthermore, we revealed that the concentrations of haptocorrin, MFGM proteins, and other bioactive proteins have never been reported in Chinese population.

The longitudinal changes in human milk bioactive proteins in Chinese population were compared with studies in other populations (Table S3). The trends we found are generally consistent with the findings in non‐Chinese populations with three minor exceptions. First, Lonnerdal et al. found the concentrations of serum albumin increased in the first 60 days of lactation and decreased thereafter, whereas we did not observe such a trend in our study. Second, Schack L et al. reported that osteopontin decreased over lactation, whereas we found that osteopontin in the first 7 postnatal days were lower than that in the 31–60 postnatal days period (Akpele & Bailey, 2004; Donovan, 2019; Greibe et al., 2013; Jiang & Lönnerdal, 2019; Liao et al., 2017; Lönnerdal et al., 2017; Nagatomo et al., 2004; Piemontese et al., 2012; Schack et al., 2009). Third, we observed a sudden increase in the lactoferrin concentration in 61–90 postnatal days, which has never been reported in other populations. However, lactoferrin levels in 61–90 postnatal days in Chinese human milk were only reported by two studies that used distinct methods and published in the year of 1986 and 2018 (Dou et al., 1986; Liu et al., 2018). Accordingly, the longitudinal change in lactoferrin in Chinese population needs to be further elucidated. Although insufficient number of studies available may cause the observed discrepancies in human milk bioactive proteins in different populations, we cannot rule out the possibility that genetic or dietary factors may cause the discrepancies. For instance, serum albumin in the blood is an indicator of the nutritional and hydration status (Akpele & Bailey, 2004). Therefore, it is entirely possible that the serum albumin in human milk is also related to maternal nutritional and hydration status.

The longitudinal changes in the bioactive proteins may have implications in infant nutrition and health. There is a sharp decrease in immunoglobulin (sIgA, IgM, and IgG) concentrations after the first 14 postnatal days, indicating that during the first 14 days of life, infants require their mothers to produce antibodies for the protection against pathogens. In this way, mothers may endow their infants with adaptive immune responses via human milk and protect them from pathogens that both the mothers and infants are exposed to (Levy, 2007). After 2 weeks of life, infants may be able to produce sufficient antibodies on their own and become less dependent on human milk for immunoglobulins (Gao et al., 2012). Additionally, the increase in the lysozyme concentration at the beginning of the 8–14 postnatal day stage suggests an increasing involvement of lysozyme in the protection of the infants against pathogenic bacteria.

This study was only able to reveal the longitudinal changes in 11 bioactive proteins. Previously, Lonnerdal et al. retrieved 7 bioactive proteins in their systematic review (Lönnerdal et al., 2017). Most of these proteins were quantified by immunoassays (Table 3), which were dependent on the development of specific antibodies. Moreover, the specificity and sensitivity of the antibodies used could contribute to variations among different studies. It should be noticed that more than 1,600 proteins have been identified in human milk due to the advances in mass spectrometry (Beck et al., 2015). In the future, mass spectrometry‐based proteomic analysis of human milk may be used to shed light on the longitudinal changes of many more bioactive proteins in human milk (Cao et al., 2017).

There are limitations in our study. First, the association between the means of delivery and the bioactive protein levels in human milk remains controversial (Affolter et al., 2016; Liu et al., 2019; Yang et al., 2018). Unfortunately, only three of the included studies specified the means of delivery, making it impossible for us to extract data and analyze the association between the means of delivery and the bioactive protein concentrations in human milk. Second, it is well‐known that premature infants are more prone to infections and that human milk decreases the rate of infections (Patel & Kim, 2018). Therefore, it is possible that milk from mothers who delivered premature infants may contain higher concentrations of immunomodulatory proteins such as immunoglobulins and lactoferrin (Bernloehr et al., 2016). Nevertheless, 20 out of the 32 identified article reported milk from mothers who delivered full‐term infants, whereas the rest did not specify the gestation age at delivery. Therefore, it is impossible for us to analyze differences in bioactive protein levels between milk from mothers delivered at different gestation ages.

## 5 CONCLUSIONS

This systematic review aimed at investigating the longitudinal changes in bioactive proteins in the human milk of the Chinese population. Data from 20 and 12 publications that were originally published in Chinese and English, respectively, were combined. The concentrations of α‐lactalbumin, lactoferrin, β‐casein, and three immunoglobulins (sIgA, IgM, and IgG) decrease during lactation. Particularly, sharp decreases are evident in the immunoglobulin concentrations during the first 14 postnatal days. Conversely, the lysozyme concentrations increase during lactation. The patterns of longitudinal changes in serum albumin, osteopontin, BSSL, and κ‐casein are less conclusive, mainly due to the limited data available. This study represents the most comprehensive report on the bioactive proteins in the human milk of the Chinese population to date.

The findings in the Chinese population are similar to those in other populations. Furthermore, it is revealed that future studies should document factors such as means of delivery, gestational age at delivery, and the protocol of milk collection to examine the association between these factors and the bioactive protein concentrations in human milk. Additionally, mass spectrometry‐based analysis of human milk proteomics may be used to investigate the longitudinal changes in many more bioactive proteins.

## 7 CONFLICT OF INTEREST

The authors declare no conflict of interest.

## 8 ETHICAL APPROVAL

This study does not involve any human or animal testing.

9

## Supporting information

Supplementary Material

## Data Availability

The data that support the findings of this study are available on request from the corresponding author.
