# Supplementary material for: Longitudinal changes in the bioactive proteins in human milk of the Chinese population: A systematic review
Source: Food Sci Nutr. 2020 Dec 19;9(1):25–35. doi: 10.1002/fsn3.2061 (PMC7802555; doi:10.1002/fsn3.2061)
Supplement: Supplementary file 1 — Supplementary Material [file FSN3-9-25-s001.doc]

Supplemental data

Longitudinal changes in the bioactive proteins in human milk of the Chinese population: A systematic review

Supplemental Table 1. Quality assessment of the included studies.

| **Reference** | **Q1** | **Q2** | **Q3** | **Q4** | **Q5** | **Q6** | **Q7** | **Q8** | **Q9** | **Q10** | **Q11** | **Q12** |
| --- | --- | --- | --- | --- | --- | --- | --- | --- | --- | --- | --- | --- |
| (Dai & Guan, 1985) | Y | NR | Y | Y | Y | CD | N | Y | NR | Y | Y | N |
| (Wang et al., 2012) | Y | NR | Y | Y | Y | CD | N | Y | NR | Y | Y | Y |
| (Wang, 2012) | Y | NR | Y | Y | Y | CD | N | Y | NR | N | Y | N |
| (Dou, Chen, & Dai, 1986) | Y | NR | Y | Y | Y | CD | N | Y | NR | Y | Y | N |
| (Min, 1989) | Y | NR | Y | Y | Y | CD | N | Y | NR | Y | Y | N |
| (Li, Guo, & Guo, 1995) | Y | Y | Y | Y | Y | CD | N | Y | NR | N | Y | N |
| (Chen, 2007) | Y | NR | Y | Y | Y | CD | N | Y | NR | N | Y | N |
| (Liu, Zheng, & Wang, 1994) | Y | Y | Y | Y | Y | CD | N | Y | NR | Y | Y | N |
| (Li, Mei, & Tang, 1995) | Y | Y | Y | Y | Y | CD | N | Y | NR | Y | Y | N |
| (Wang & Lin, 1997) | NR | NR | Y | Y | Y | CD | N | Y | NR | N | Y | N |
| (Wang, 1987) | NR | NR | Y | Y | NR | CD | N | Y | NR | N | Y | N |
| (Liu & Zhang, 2005) | Y | NR | Y | Y | Y | CD | N | Y | NR | N | Y | N |
| (Wu, Li, & Chen, 1995) | Y | NR | Y | Y | Y | CD | N | Y | NR | Y | Y | N |
| (Wei & Pan, 1991) | NR | NR | Y | Y | Y | CD | N | Y | NR | N | Y | N |
| (Liu, Dong, & Jiang, 2018) | NR | NR | Y | Y | Y | CD | N | Y | Y | Y | Y | Y |
| (Jiang, 2017) | NR | NR | Y | Y | Y | CD | Y | Y | Y | N | Y | N |
| (Shan, Wang, & Chen, 2011) | Y | NR | Y | Y | Y | CD | N | Y | Y | Y | Y | N |
| (Jiang, Fu, & Yan, 1999) | Y | NR | Y | Y | Y | CD | N | Y | NR | N | Y | N |
| (Chen, Zheng, & Qian, 1986) | Y | NR | Y | Y | Y | Y | N | Y | NR | Y | Y | N |
| (Han, Pang, & Liu, 2010) | NR | NR | Y | Y | Y | CD | N | Y | NR | N | Y | Y |
| (Jackson et al., 2004) | Y | NR | Y | Y | Y | CD | N | Y | NR | N | Y | Y |
| (Hsu et al., 2014) | Y | NR | Y | Y | Y | CD | N | Y | NR | N | Y | Y |
| (Liu, Gu, Ye, Ren, & Guo, 2019) | NR | NR | Y | Y | Y | CD | N | Y | NR | Y | Y | Y |
| (Yang et al., 2018) | Y | NR | Y | Y | Y | CD | N | Y | Y | Y | Y | Y |
| (Urwin et al., 2013) | NR | NR | Y | Y | Y | CD | N | Y | Y | Y | Y | Y |
| (Cai et al., 2018) | NR | NR | Y | Y | Y | CD | Y | Y | NR | N | Y | Y |
| (Yuen, Loke, & Gohel, 2012) | Y | NR | Y | Y | Y | CD | N | Y | NR | N | Y | Y |
| (Bruun et al., 2018) | NR | NR | Y | Y | Y | CD | N | Y | NR | N | Y | Y |
| (Affolter et al., 2016) | Y | NR | Y | Y | Y | Y | N | Y | Y | Y | Y | Y |
| (Shi et al., 2011) | NR | NR | Y | Y | Y | CD | N | Y | NR | N | Y | N |
| (Sha, Zhou, Xi, Li, & Li, 2019) | NR | NR | Y | Y | Y | CD | N | Y | NR | Y | Y | Y |
| (Elwakiel et al., 2019) | Y | NR | Y | Y | Y | CD | N | Y | NR | N | Y | Y |

Note:

Q1: Did the authors clearly describe that the infants were full-term or pre-term?

Q2: Did the authors describe whether births were via vaginal delivery or C-section?

Q3: Did the authors clearly describe that the mothers were healthy?

Q4: Did the authors clearly describe the number of mothers recruited in the study?

Q5: Did the authors clearly describe the geographic locations of human milk sample collection?

Q6: Did the authors clearly describe whether the human samples were collected from urban or rural areas?

Q7: Did the authors study the dietary intake of lactating mothers and provide related data?

Q8: Did the authors clearly describe the lactation stage without using the terms colostrum, transition milk, or mature milk?

Q9: Did the authors clearly describe the foremilk, hindmilk, full expression, or pooled full expression within 3-4 hours?

Q10: Did the authors clearly describe the time of day when the human milk was collected or whether all the human milk was collected at the same time of the day?

Q11: Did the authors provide detailed methods for measuring the bioactive protein concentrations?

Q12: Did the authors describe the funding sources (for-profit organizations, non-profit funding, or government funding) and conflicts of interests of the authors?

CD, cannot be determined; NR, not reported; N, no; Y, yes.

**Supplemental Table 2. The longitudinal changes in the protein concentrations and the percentages of the total proteins of the bioactive proteins in the human milk of the Chinese population.**

| **Bioactive protein** | **1-7 d** | | | **8-14 d** | | | **15-30 d** | | | **31-60 d** | | | **61-90 d** | | | **91-365 d** | | |
| --- | --- | --- | --- | --- | --- | --- | --- | --- | --- | --- | --- | --- | --- | --- | --- | --- | --- | --- |
|  | **n** | **Mean ± SD (****mg/100 mL)** | **Proportion %** | **n** | **Mean ± SD (mg/100 mL)** | **Proportion %** | **n** | **Mean ± SD (mg/100 mL)** | **Proportion %** | **n** | **Mean ± SD (mg/100 mL)** | **Proportion %** | **n** | **Mean ± SD (mg/100 mL)** | **Proportion %** | **n** | **Mean ± SD (mg/100 mL)** | **Proportion %** |
| α-lactalbumin | 224 | 377.14±58.90^a^ | 17.71±2.77^e^ | 240 | 335.99±6.98^b^ | 19.31±0.40^d^ | 90 | 316.00±0.00^c^ | 19.75±0.00^c^ | 300 | 300.24±18.18^d^ | 22.75±1.38^a^ | - | - | - | 90 | 228.00±0.00^e^ | 20.92±0.00^b^ |
| Lactoferrin | 464 | 268.11±69.97^a^ | 12.59±3.29^b^ | 330 | 215.52±70.23^b^ | 12.39±4.04^b^ | 161 | 162.82±26.41^d^ | 10.18±1.65^c^ | 217 | 125.29±9.02^e^ | 9.49±0.68^d^ | 70 | 175.71±70.53^c^ | 15.41±6.19^a^ | 352 | 99.06±23.22^f^ | 9.09±2.13^d^ |
| Serum albumin | 74 | 29.40±20.59^c^ | 1.4±0.97^e^ | 90 | 48.00±0.00^a^ | 2.76±0.00^c^ | 90 | 48.00±0.00a | 3±0.00^b^ | 60 | 29.40±0.00^c^ | 2.23±0.00^d^ | - | - | - | 90 | 42.00±0.00^b^ | 3.85±0.91^a^ |
| sIgA | 2089 | 331.67±140.30^a^ | 15.57±6.59^a^ | 334 | 113.20±62.05^b^ | 6.51±3.56^b^ | 291 | 57.58±11.34^c^ | 3.6±0.71^c^ | 305 | 61.42±30.70^c^ | 4.65±2.32^c^ | 63 | 24.31±2.53^d^ | 2.13±0.23^d^ | 215 | 38.43±15.50^cd^ | 3.53±1.42^c^ |
| IgM | 962 | 42.03±35.75^a^ | 1.97±1.68^a^ | 99 | 11.99±0.92^b^ | 0.69±0.05^b^ | 179 | 4.58±2.97^c^ | 0.29±0.19^c^ | 215 | 2.77±1.10^c^ | 0.21±0.08^c^ | 63 | 0.98±0.18^c^ | 0.09±0.02^c^ | 189 | 1.74±0.88^c^ | 0.16±0.08^c^ |
| IgG | 1046 | 37.08±36.12^a^ | 1.74±1.70^a^ | 99 | 2.31±0.34^b^ | 0.14±0.02^b^ | 179 | 4.55±2.32^b^ | 0.28±0.15^b^ | 215 | 6.11±4.93^b^ | 0.46±0.37^b^ | 63 | 4.64±2.01^b^ | 0.41±0.18^b^ | 215 | 2.85±1.50^b^ | 0.26±0.14^b^ |
| Lysozyme | 109 | 10.09±4.45^c^ | 0.6±0.33^d^ | 32 | 7.71±2.04^d^ | 0.44±0.11^e^ | 156 | 9.63±2.30^c^ | 0.6±0.14^d^ | 79 | 9.89±1.08^c^ | 0.75±0.08^c^ | 68 | 11.19±0.91^b^ | 0.98±0.08^b^ | 227 | 13.58±1.30^a^ | 1.25±0.12^a^ |
| Osteopontin | 7 | 17.00±0.00 | 0.80±0.00 | - | - | - | - | - | - | 76 | 26.62±0.00 | 2.02±0.00 | - | - | - | - | - | - |
| BSSL | 81 | 9.64±0.00 | 0.45±0.00 | - | - | - | - | - | - | - | - | - | - | - | - | 67 | 19.88±0.00 | 1.82±0.00 |
| β-Casein | 217 | 333.97±107.85^a^ | 15.7±5.06^c^ | 150 | 296.08±0.00^b^ | 17.02±0.00^b^ | - | - | - | 210 | 310.52±71.31^b^ | 23.52±5.40^a^ | - | - | - | - | - | - |
| κ-Casein | 67 | 41.72±14.78 | 1.96±0.30 | 60 | 23.1±0.00 | 1.75±0.00 | - | - | - | - | - | - | - | - | - | - | - | - |

Note: Means with different letters were significantly different (*p*<0.05 one-way ANOVA followed by the Student-Newman-Keuls test).

**Supplemental Table 3**. Selected studies of the longitudinal changes in bioactive protein concentrations in the human milk of the non-Chinese population.

| Reference | Study type | Country | Bioactive protein | 0-5 d | | 6-15 d | | 16-30 d | | 31-60 d | | 61-90 d | | 91-365 d | |
| --- | --- | --- | --- | --- | --- | --- | --- | --- | --- | --- | --- | --- | --- | --- | --- |
|  |  |  |  | mg/100 mL | % | mg/100 mL | % | mg/100 mL | % | mg/100 mL | % | mg/100 mL | % | mg/100 mL | % |
| (Akpele & Bailey, 2004; Donovan, 2019; Lönnerdal, Erdmann, Thakkar, Sauser, & Destaillats, 2017) | Systematic review | Sweden, Ethiopia，Spain，Argentina，Japan，France，Peru，Pakistan，USA | α-lactalbumin | 456 | 22.14 | 430 | 25.29 | 352 | 22.43 | 310 | 23.85 | 284 | 23.67 | 262 | 23.82 |
|  |  |  | Lactoferrin | 615 | 29.85 | 365 | 21.47 | 246 | 15.68 | 195 | 15.00 | 189 | 15.75 | 144 | 13.09 |
|  |  |  | Serum albumin | 35 | 1.70 | 62 | 3.65 | 67 | 4.27 | 69 | 5.31 | 45 | 3.75 | 37 | 3.36 |
|  |  |  | sIgA | 545 | 26.46 | 150 | 8.82 | 110 | 7.01 | 100 | 7.69 | 130 | 10.83 | - | - |
|  |  |  | IgM | - | - | 12 | 0.71 | 5 | 0.32 | - | - | 3 | 0.25 | 3 | 0.27 |
|  |  |  | IgG | - | - | 5 | 0.29 | 5 | 0.32 | - | - | 3 | 0.25 | 4 | 0.36 |
|  |  |  | Lysozyme | 32 | 1.55 | 30 | 1.76 | 28 | 1.78 | 110 | 8.46 | 85 | 7.08 | - | - |
| (Donovan, 2019; Jiang & Lönnerdal, 2019; Nagatomo, Ohga, Takada, Nomura, & Hara, 2004; Schack et al., 2009) | Original | Denmark, Japan, USA | Osteopontin | 17.8 | 0.86 | 13.1 | 0.77 | 11.44 | 0.73 | 9.63 | 0.74 | - | - | 5.1 | 0.46 |
| (Liao et al., 2017) | Original | USA | β-Casein | 125.4 | 6.09 | 139.89 | 8.23 | 136 | 8.66 | 120 | 9.23 | 127 | 10.58 | 95.2 | 8.65 |
| (Liao et al., 2017) | Original | USA | κ-Casein | 85.6 | 4.16 | 83 | 4.88 | 73 | 4.65 | 59 | 4.54 | 62 | 5.17 | 52.6 | 4.78 |

Affolter, M., Garciarodenas, C. L., Vinyespares, G., Jenni, R., Roggero, I., Avantinigro, O., . . . Wang, P. (2016). Temporal changes of protein composition in breast milk of Chinese urban mothers and impact of caesarean section delivery. *Nutrients, 8*(8), 504. doi:10.3390/nu8080504

Akpele, L., & Bailey, J. L. (2004). Nutrition counseling impacts serum albumin levels. *Journal of Renal Nutrition, 14*(3), 143-148. doi:10.1053/j.jrn.2004.04.003

Bruun, S., Jacobsen, L. N., Ze, X., Husby, S., Ueno, H. M., Nojiri, K., . . . Yan, S. (2018). Osteopontin levels in human milk vary across countries and within lactation period: data from a multicenter study. *Journal of Pediatric Gastroenterology and Nutrition, 67*(2), 250-256. doi:10.1097/MPG.0000000000002004

Cai, X., Duan, Y., Li, Y., Wang, J., Mao, Y., Yang, Z., . . . Yin, S. (2018). Lactoferrin level in breast milk: a study of 248 samples from eight regions in China. *Food & Function, 9*(8), 4216-4222. doi:10.1039/C7FO01559C

Chen, B. (2007). Study on the anti-infection effect of sIgA in breast milk. *ChongQing Medical University*.

Chen, Y., Zheng, D., & Qian, Y. (1986). Dynamic observation on the milk sIgA of premature and full-term infants. . *Chinese Journal of Neonatology, 1*(5), 221-224.

Dai, Q., & Guan, H. (1985). Dynamic observation of immune bodies in the breast milk and serum of postpartum mother. *Acta Universitatis Medictnae Tangji*.

Donovan, S. M. (2019). Human milk proteins: composition and physiological significance. *Karger Publishers, 90*(93-101). doi:10.1159/000490298

Dou, G., Chen, M., & Dai, W. (1986). Dynamic observation of lactoferrin, lysozyme, C3 and immunoglobulin in human milk in different lactation *Shanghai Journal of Immunology. 1986; 6(2):98-100, 6*(2), 98-100.

Elwakiel, M., Boeren, S., Hageman, J. A., Szeto, I. M., Schols, H. A., & Hettinga, K. (2019). Variability of serum proteins in Chinese and Dutch human milk during lactation. *Nutrients, 11*(3), 499. doi:10.3390/nu11030499

Han, L., Pang, K., & Liu, A. (2010). Determination of nutritional and bioactive components of woman colostrum in Zhengzhou city. . *Journal of Zhengzhou University (Medical Sciences), 45*(1), 59-61. doi:10.3969/j.issn.1671-6825.2010.01.019

Hsu, Y. C., Chen, C. H., Lin, M. C., Tsai, C. R., Liang, J. T., & Wang, T. M. (2014). Changes in preterm breast milk nutrient content in the first month. *Pediatrics and Neonatology, 55*(6), 449-454. doi:10.1016/j.pedneo.2014.03.002

Jackson, J. G., Janszen, D. B., Bo, L., Lien, E. L., Pramuk, K. P., & Kuhlman, C. F. (2004). A multinational study of α-lactalbumin concentrations in human milk. *Journal of Nutritional Biochemistry, 15*(9), 517-521. doi:10.1016/j.jnutbio.2003.10.009

Jiang, J. (2017). *Study on important nutrients, bioactive factors and small molecule metabolites in breast milk.* (Master), Zhejiang University, Hangzhou, Beijing.

Jiang, J., Fu, F., & Yan, Y. (1999). Compare the concentrations of anti-infective factors of human colostrum between cesarean section and normal labor. *Guangdong Medical Journal, 20*(4), 254-255. doi:10.13820/j.cnki.gdyx.1999.04.009

Jiang, R., & Lönnerdal, B. (2019). Osteopontin in human milk and infant formula affects infant plasma osteopontin concentrations. *Pediatric Research, 85*(4). doi:10.1038/s41390-018-0271-x

Li, L., Guo, B., & Guo, Z. (1995). Determination of iron, copper, total protein, fat, immunoglobulin in colostrum. *Journal of Lanzhou Medical College, 21*(4), 221. doi:10.13885/j.issn.1000-2812.1995.04.014

Li, S., Mei, Z., & Tang, Z. (1995). Observation on the content of immunoglobulin and complement in breast milk. *Journal of Practical Obstetrics and Gynecology, 11*(6), 308.

Liao, Y., Weber, D., Xu, W., Durbin-Johnson, B. P., Phinney, B. S., & L?Nnerdal, B. (2017). Absolute quantification of human milk caseins and the whey/casein ratio during the first year of lactation. *Journal of Proteome Research*, 4113. doi:10.1021/acs.jproteome.7b00486

Liu, B., Gu, F., Ye, W., Ren, Y., & Guo, S. (2019). Colostral and mature breast milk protein compositional determinants in Qingdao, Wuhan and Hohhot: maternal food culture, vaginal delivery and neonatal gender. *Asia Pacific Journal of Clinical Nutrition, 28*(4), 800.

Liu, M., & Zhang, H. (2005). Determination and clinical significance of immunoglobulin in breast milk. *Chinese Journal of Practical Gynecology and Obstetrics, 21*(6), 324-324. doi:10.3969/j.issn.1005-2216.2005.06.029

Liu, R., Zheng, D., & Wang, M. (1994). Observation on the content of immunoglobulin in breast milk. *Chinese Journal of Applied Clinical Pediatrics*(3).

Liu, Y., Dong, X., & Jiang, T. (2018). Lactoferrin concentration in human milk and its affection factors. *China Food Additives*(8), 70-74.

Lönnerdal, B., Erdmann, P., Thakkar, S. K., Sauser, J., & Destaillats, F. (2017). Longitudinal evolution of true protein, amino acids and bioactive proteins in breast milk: a developmental perspective. *Journal of Nutritional Biochemistry, 41*, 1-11. doi:10.1016/j.jnutbio.2016.06.001

Min, X. (1989). Analysis of immune substances in early maternal milk. *Shanghai Journal of Immunology, 9*(1), 29-31.

Nagatomo, T., Ohga, S., Takada, H., Nomura, A., & Hara, T. (2004). Microarray analysis of human milk cells: persistent high expression of osteopontin during the lactation period. *Clinical and Experimental Immunology, 138*(1). doi:10.1111/j.1365-2249.2004.02549.x

Schack, L., Lange, A., Kelsen, J., Agnholt, J., Christensen, B., Petersen, T. E., & S?Rensen, E. S. (2009). Considerable variation in the concentration of osteopontin in human milk, bovine milk, and infant formulas. *Journal of Dairy Science, 92*(11), 0-5385. doi:10.3168/jds.2009-2360

Sha, L., Zhou, S., Xi, Y., Li, R., & Li, X. (2019). The level of bile salt-stimulated lipase in the milk of Chinese women and its association with maternal BMI. *Journal of Biomedical Research*, 1-7. doi:10.7555/JBR.33.20180107

Shan, J., Wang, X., & Chen, X. (2011). Preliminary determination of lactoferrin in human breast milk. *J Clin Pediatr, 29*(6), 549-551. doi:10.3969/j.issn.1000-3606.2011.06.013.

Shi, Y., Sun, G., Zhang, Z., Deng, X., Kang, X., Liu, Z., . . . Sheng, Q. (2011). The chemical composition of human milk from Inner Mongolia of China. *Food Chemistry, 127*(3), 1193-1198. doi:10.1016/j.foodchem.2011.01.123

Urwin, H. J., Zhang, J., Gao, Y., Wang, C., Li, L., Song, P., . . . Miles, E. A. (2013). Immune factors and fatty acid composition in human milk from river/lake, coastal and inland regions of China. *British Journal of Nutrition, 109*(11), 1949-1961. doi:10.1017/s0007114512004084

Wang, G. X., Zhao, H. M., Zhu, X. X., Yang, X. M., Wang, H. Y., Huang, Q. T., . . . Jing, L. I. (2012). Project of hospital education in lactation practices improves breast milk siga concentration. *Journal of Clinical Pediatrics*.

Wang, J. (2012). Impact of differnet delivery ways on colostrum lctation time and immunoglobulin a level. *China Tropical Medicine, 12*(12), 1519-1521. doi:10.13604/j.cnki.46-1064/r.2012.12.008

Wang, M. (1987). Determination of immunoglobulin in breast milk. *Central Plains Medical Journal.*(6), 36-37.

Wang, Y., & Lin, Q. (1997). Lysozyme in breast milk and physiological diarrhea in infants. *Chinese Journal of Microecology, 9*(5), 32-33,35. doi:10. 13381 /j.cnki.cjm.1997.05.010

Wei, Z., & Pan, X. (1991). Determination of lysozyme in breast milk. *Acta Universitatis Medicinalis Nanjing, 11*(1), 16-18.

Wu, C., Li, X., & Chen, W. (1995). Determination and analysis of immunoglobulin and complement content in breast milk. *Chinese Journal of Birth Health & Heredity, 3*(6), 91,94. doi:10.13404 /j.cnki.cjbhh.1995.06.042

Yang, Z., Jiang, R., Chen, Q., Wang, J., Duan, Y., Pang, X., . . . Lonnerdal, B. (2018). Concentration of Lactoferrin in Human Milk and Its Variation during Lactation in Different Chinese Populations. *Nutrients, 10*(9), 1235. doi:10.3390/nu10091235

Yuen, J. W. M., Loke, A. Y., & Gohel, M. I. (2012). Nutritional and immunological characteristics of fresh and refrigerated stored human milk in Hong Kong: a pilot study. *Clinica Chimica Acta, 413*(19), 1549-1554. doi:10.1016/j.cca.2012.03.018
